# Community Paramedicine Intervention Reduces Hospital Readmission and Emergency Department Utilization for Patients with Cardiopulmonary Conditions

**DOI:** 10.5811/westjem.57862

**Published:** 2023-07-10

**Authors:** Aaron Burnett, Sandi Wewerka, Paula Miller, Ann Majerus, John Clark, Landon Crippes, Tia Radant

**Affiliations:** * Regions Hospital, St. Paul, Minnesota; † Critical Care Research Center, Regions Hospital, St. Paul, Minnesota; ‡ St. Paul Fire Department, St. Paul, Minnesota

## Abstract

**Objective:**

Patients discharged from the hospital with diagnoses of myocardial infarction, congestive heart failure or acute exacerbation of chronic obstructive pulmonary disease (COPD) have high rates of readmission. We sought to quantify the impact of a community paramedicine (CP) intervention on hospital readmission and emergency department (ED) and clinic utilization for patients discharged with these conditions and to calculate the difference in healthcare costs.

**Methods:**

This was a prospective, observational cohort study with a matched historical control. The groups were matched for qualifying diagnosis, age, gender, and ZIP code. The intervention group received 1–2 home visits per week by a community paramedic for 30 days. We calculated the number of all-cause hospital readmissions and ED and clinic visits, and used descriptive statistics to compare cohorts.

**Results:**

Included in the study were 78 intervention patients and 78 controls. Compared to controls, fewer subjects in the CP cohort had experienced a readmission at 120 days (34.6% vs 64.1%, *P* < 0.001) and 210 days (43.6% vs 75.6%, *P* < 0.001) after discharge. At 210 days the CP cohort had 40.9% fewer total hospital admissions, saving 218 bed days and $410,428 in healthcare costs. The CP cohort had 40.7% fewer total ED visits.

**Conclusion:**

Patients who received a post-hospital community paramedic intervention had fewer hospital readmissions and ED visits, which resulted in saving 218 bed days and decreasing healthcare costs by $410,428. Incorporation of a home CP intervention of 30 days in this patient population has the potential to benefit payors, hospitals, and patients.

## INTRODUCTION

### Problem

We designed this investigation to quantify the impact of a one-month community paramedic (CP) intervention on all-cause hospital readmissions and visits to emergency departments (ED) and clinics at 30, 120 and 210 days post-hospital discharge for patients with congestive heart failure (CHF), acute myocardial infarction (AMI) and chronic obstructive pulmonary disease (COPD) as compared to a matched control group. We also quantified the difference in total cost of care between cohorts over the data collection period. Our hypothesis was that a CP intervention would decrease the number of all-cause readmissions, ED visits, and total cost of care, and increase clinic utilization.

### Background

Patients readmitted to the hospital often have poor outcomes and incur high healthcare costs.^1^ In addition, patient satisfaction scores are significantly and negatively correlated with the hospital’s 30-day readmission rates for AMI, CHF, and pneumonia (PNA).^2^ Patients with CHF have up to a 25% chance of being readmitted to the hospital within 30 days,^3^,^4^ while those odds are nearly 20% in patients with AMI^5^ and 15% in patients with exacerbations of COPD.^6^ Moreover, the majority of readmissions for patients with AMI, CHF, or PNA occur within the first two weeks of hospital discharge.^7^ Reducing hospital readmissions and repeat ED visits for patients with common chronic conditions is a key feature of healthcare reform efforts for both clinicians and payors.

As part of the Hospital Readmissions Reduction Program, Medicare payments have been reduced up to 3% to hospitals with excess readmissions for six conditions, including CHF, AMI, and COPD, since 2012.^8^ Predictive modeling has been used to identify factors (eg, age, socioeconomic status, primary language, multiple medications, and place of residence) that put patients with CHF, AMI and COPD at highest risk for readmission.^9^ Studies that have looked at the ability of predictive modeling to identify patients with acute cardiopulmonary diagnoses who are at highest risk of 30-day readmission vary greatly in their conclusions. No consensus has been reached on interventions to target the highest risk group.^10^

With varying rates of success, hospitals have implemented numerous approaches to reduce readmissions including single and bundled strategies, improved care coordination, better hospital discharge education, medication reconciliation, scheduling of follow-up visits before discharge, and standardization of communication tools at discharge.^11^–^19^ Identification of a minimum threshold bundle of interventions proven to reduce readmissions remains elusive. Patients report feeling unprepared for discharge; the primary reasons for readmission include having difficulty performing activities of daily living, having problems adhering to or accessing medications, and lack of social support.^20^

One intervention that has been proposed to decrease hospital readmissions is the inclusion of a community paramedic in the outpatient care plan. Community paramedics are advanced, subspecialized paramedics who have undergone additional training designed to transition the application of their Advanced Life Support skillset from the emergency setting to the primary care setting. Community paramedicine takes advantage of the knowledge base and mobile capabilities of paramedics and leveragess their ability to conduct in-home assessments. Prior work with CPs has been reported, however, published research on the impact of CPs on ED utilization and hospital readmissions for patients with AMI, COPD or CHF is scarce. Prior published studies on community paramedicine have demonstrated generally positive results but have mainly focused on reducing 911 calls and ED usage.

**Population Health Research Capsule**

What do we already know about this issue?

*Patients discharged from the hospital with diagnoses of myocardial infarction, congestive heart failure, or chronic obstructive pulmonary disease have high rates of readmission*.

What was the research question?

*Can a community paramedic intervention reduce hospital and emergency department (ED) readmission in this patient population?*

What was the major finding of the study?

*Community paramedic (CP) intervention reduced readmissions at 120 (34.6% vs 64.1%, P* < *0.001) and 210 days (43.6% vs 75.6%, P* < *0.001)*

How does this improve population health?

*Implementation of a CP program decreases the need for re-hospitalization and decreases healthcare costs in this patient population*.

### Purpose

The main limitation to the integration of CPs into the current healthcare model as a recognized discipline is the paucity of safety, efficacy, and long-term outcomes data to demonstrate their impact on patient care. Our goal in this study was to contribute to this dialogue by describing the impact of a CP intervention on 210-day hospital readmission and ED and clinic utilization in patients discharged from an acute hospitalization with AMI, CHF, or an acute exacerbation of COPD as compared to a matched control group. Our project is the first to examine the impact of a CP intervention by using a prospective, observational cohort with a matched historical control. We followed patients for 210 days after discharge (180 days after completion of the CP intervention) in an attempt to better explore the impact of the intervention on the patient’s longer term health.

## METHODS

### Design and Setting

This project was reviewed by our organization’s Research Subjects Protection Program office and was granted a waiver from ongoing oversight from the institutional review board. The local urban fire/emergency medical services (EMS) agency, in collaboration with the local Level I trauma center, created a CP program in 2014 staffed by certified community paramedics. The fire department employed the community paramedics, and medical direction was provided by an EMS board-certified physician from the trauma center.

### Selection of Participants

In January 2015, we began to prospectively identify patients admitted to the hospital for AMI, COPD or CHF. Participants met the following inclusion criteria: 1) ≥18 years old; 2) lived in the same city where the hospital is located; 3) discharged from an acute admission with a diagnosis of CHF, AMI, or COPD; 4) was not eligible for traditional home healthcare; and 5) was referred by an in-patient treatment team. Exclusion criteria were as follows: 1) non–English-speaking; 2) planning a move outside city limits in the 60 days after enrollment; and 3) being a prisoner. Participants were approached by the research team and offered participation after it was determined they met the inclusion criteria and their treating clinician had placed a referral for the intervention.

A historical control cohort was matched on age, gender, qualifying diagnosis, and ZIP code of residence. We retrospectively obtained data regarding control patients from a group of patients admitted to the hospital during the same data collection period who met eligibility criteria but did not participate in the intervention. Control group patients were not offered the intervention nor were they referred for the intervention; however, they met all other enrollment criteria. If a control patient had multiple hospital visits during the search period, one of the visits was chosen at random.

### Interventions

**Figure 1:**
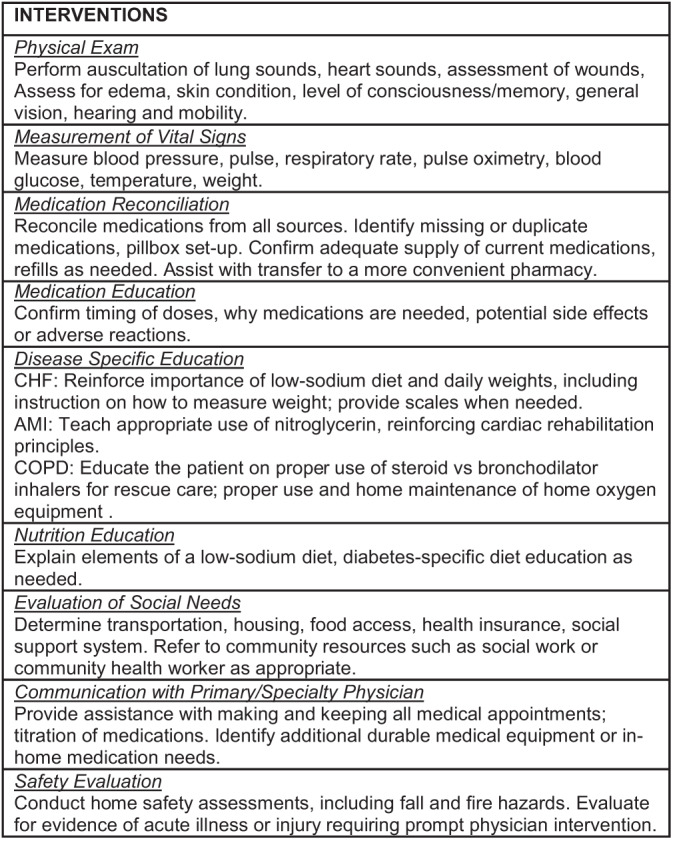
Standardized elements of community paramedic visits. *AMI*, acute myocardial infarction; *COPD*, chronic obstructive pulmonary disease; CHF, congestive heart failure.

Patients were enrolled in the CP intervention prior to hospital discharge. When possible, and with few exceptions, the patient and the CP met during enrollment while the patient was still hospitalized. Upon discharge the CP visited the patient in their home within 48 hours and subsequently 1–2 times per week for 30 days. While the home visit contained standardized elements (Figure 1), the CPs were allowed to individualize how they prioritized the specific required elements based on their needs assessment for each patient.

The CPs were granted access to the hospital electronic health record (EHR) for review of patient medical history, but they completed documentation of each visit in the ambulance service electronic patient care report (HealthEMS, Stryker Corp, Redmond, WA). Physician supervision of the CP intervention was provided by the ambulance service’s EMS medical director in partnership with the patient’s primary care team. Communication with the primary care physician typically occurred via a telephone call to the registered nurse at the clinic who would relay information to the physician. Patient satisfaction surveys were administered by a research staff member at the final home visit.

### Measurements

We used manual review of the EHR to quantify healthcare utilization for this project. Using the Care Everywhere function of the EHR, staff were able to identify healthcare visits within our integrated care delivery system and within most of the other local healthcare systems.

**Figure 2:**
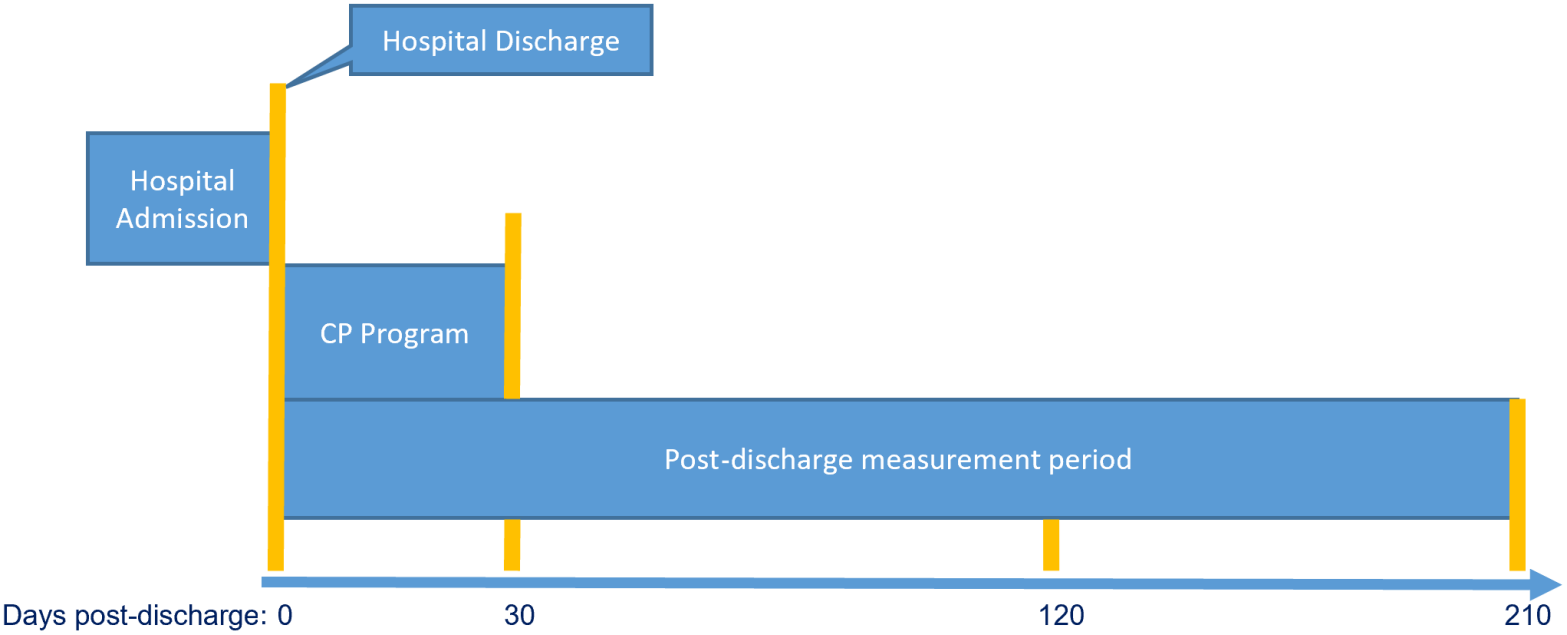
Program timeline. *CP*, community paramedicine.

Patient demographics are summarized using descriptive statistics in Table 1. We calculated healthcare utilization (admissions, ED visits, and clinic visits) by cohort at 30 days (at the conclusion of the CP intervention), 120 days, and 210 days post-hospital discharge for both groups (Figure 2). We compared the percentage of subjects by type and time point of utilization using chi-square tests (Table 2).

We computed the median utilization count per subject and generated an incidence rate ratio (IRR) using Poisson regression (Table 3). Total utilization count by type and time point for each cohort was also computed (Table 4). We calculated healthcare savings by comparing actual costs to the healthcare system for the CP group and the control group. Average cost to the program per CP home visit was $100 inclusive of CP salary, vehicle use, fuel and supply expenses, and physician medical direction. Patients received an average of five CP visits. Average cost per hospital admission was $6,413.00 for an average length of stay of four days based on the hospital’s average for the diagnoses included in the intervention.

## RESULTS

### Findings

The intervention and control cohorts each consisted of 78 patients. There were no significant demographic differences between the CP and control groups (Table 1). Compared to controls, fewer subjects in the CP cohort had experienced a readmission at 120 days and 210 days after discharge (Table 2). A significantly higher percentage of CP subjects had at least one clinic visit in the first 30 days, although this difference was not observed at 120 or 210 days. Regression results for hospital readmissions indicated a lower likelihood of readmissions for CP subjects throughout the follow-up period compared to controls (30 days IRR 0.53; 120 days IRR 0.52; and 210 days IRR 0.59). Similarly, regression results for ED visits also indicated lower likelihood of ED utilization for CP subjects compared to controls (30 days IRR 0.50; 120 days IRR 0.54; and 210 days IRR 0.59) (Table 3). At 210 days after discharge there were 56 (40.9%) fewer hospital admissions in the CP cohort (81 vs 137), which resulted in saving 218 bed days and $410,428 in healthcare costs (Table 4). Patients in the CP cohort had 70 (40.7%) fewer ED visits (102 vs 172). Of the 78 patients in the CP cohort, 78 (100%) recommended this program to others on the exit survey.

## DISCUSSION

As healthcare systems evolve, new care delivery models are needed to control costs, improve outcomes, and increase patient satisfaction. Our data suggest that CPs can be part of the solution by decreasing hospital readmissions and ED visits for a population that has been shown to have high rates of readmission. Traditionally, post-discharge readmission studies have targeted a period of 30 days. Our project quantified differences in healthcare utilization between cohorts at 30, 120, and 210 days after hospital discharge in an attempt to better explore the impact of the one-month CP intervention on the patient’s longer term health. During this period the benefits of the CP intervention were maintained, the patients remained out of both the hospital and the ED, and the total cost of care was decreased. There were no identified increased adverse events in the intervention group. Patient surveys administered at the final paramedic visit indicated that patients overwhelmingly recommended this intervention, demonstrating that it improved the patient experience.

Establishing a permanent role for CPs in a hospital system’s readmission-avoidance processes requires that the CP program develops mechanisms to ensure financial sustainability. Our data demonstrate the cost savings that were possible with a CP intervention. In our case, the savings were not related directly to the hospital but instead were reflected in the decreased total cost of care for the insurers covering the patient’s medical costs. To obtain access to these cost savings to fund a CP program, insurers must be persuaded to cover CP visits. Our data should make these conversations more empirical and evidenced based.

While the primary cost savings demonstrated by our intervention was to the benefit of the payors, the increased availability of hospital bed days was a direct benefit to our hospital. Like many tertiary care centers, our hospital is routinely operating at 95% capacity with multiple episodes of >100% capacity per month. This in turn results in ED boarding and the potential for ambulance diversions, which have been shown to have a negative financial impact on a hospital.^21^ In this way our CP intervention offered a direct benefit to the receiving hospital; however, the financial impact to the hospital was difficult to quantify, which could lead to challenges in advocating for a hospital-financed CP program.

Our project is the first to examine the impact of a CP intervention by using a prospective, observational cohort with a matched historical control study design. By focusing on acute cardiopulmonary processes we were able to narrow our CPs’ medical assessment on the most common components of this population’s outpatient management including medications, comorbidities, and signs/symptoms of decompensation. Conducting the assessment in the patients’ homes, as opposed to the clinic, allowed our CPs an opportunity to visualize the patients’ living conditions and food resources and to have conversations about transportation, addiction, and mental health to better evaluate the social determinants of treatment failure. Many of these contributing factors were incompletely understood when the patient visited the primary care clinic, as key data that could be found only in the home were unavailable.

## LIMITATIONS

There were limitations to the study. Community paramedics have varied education and clinical experience in providing post-acute care. We were unable to identify what specific CP intervention(s) led to the decreases in observed healthcare utilization. While our project followed patients for 210 days after hospitalization and 180 days after completion of the CP intervention, we were not able to extrapolate our data past this point. Additionally, although our EHR captures healthcare utilization at many local healthcare systems, it is possible that patients included in the CP or control groups had additional visits or hospitalizations that were not included in this analysis. However, because it was unlikely that it differed between the CP and control groups, it is unlikely that it introduced bias into the primary analysis. While we were able to describe cost savings to the healthcare system for this intervention, we were not able to describe the costs associated with program start-up or ongoing programming.

## CONCLUSION

Our project demonstrated that an in-home community paramedic intervention conducted for 30 days with patients discharged from the hospital for CHF, AMI or COPD resulted in decreased hospital readmissions and decreased ED visits at 30, 120 and 210 days after hospital discharge. In addition, a savings of $410,428 for payors and an increase of 218 available hospital bed days was realized in the intervention cohort. Incorporation of a home CP intervention of 30 days in this patient population has the potential to benefit payors, hospitals and, most importantly, patients. The implications of our findings are important. As healthcare systems seek innovative approaches to reduce cost, improve quality of care, and enhance patient experience, new care models must be implemented. This project demonstrates that a 30-day, community paramedic intervention in the home for patients discharged from an acute hospitalization for CHF, AMI or COPD results in decreased hospital and ED readmissions while decreasing the total cost of care and improving hospital bed availability.

## Figures and Tables

**Table 1:** Subject characteristics at baseline.

	CP cohort (N = 78)	Control cohort (N = 78)	*P*-value
**Age** - Median (IQR)	63.5 (17.0)	64 (17.0)	0.81
**Gender -** N (%)			0.87
Male	44 (56.41%)	43 (55.13%)	
Female	34 (43.59%)	35 (44.87%)	
**Race -** N (%)			0.80
American Indian or Alaska Native	0 (0%)	1 (1.28%)	
Asian	1 (1.28%)	1 (1.28%)	
Black or African-American	23 (29.49%)	24 (30.77%)	
Hispanic or Latino	1 (1.28%)	1 (1.28%)	
Native Hawaiian or other	0 (0%)	1 (1.28%)	
White	52 (66.67%)	50 (64.10%)	
Other	1 (1.28%)	0 (0%)	
**Qualifying diagnosis**			
Congestive heart failure			
Chronic obstructive pulmonary disease			
Acute myocardial infarction			

*IQR*, interquartile range.

**Table 2:** Cumulative percentage of patients in each cohort who had at least one hospital readmission, emergency department visit, or clinic visit at each study time point.

Days post discharge	CP cohort (N = 78)	Control cohort (N = 78)	*P*-value*
Hospital readmissions			
30	20.5%	32.1%	0.10
120	34.6%	64.1%	<0.001*
210	43.6%	75.6%	<0.001*
ED visits			
30	24.4%	37.2%	0.08
120	42.3%	65.4%	<0.01*
210	52.6%	79.5%	<0.001*
Clinic visits			
30	92.3%	70.5%	<0.001*
120	93.6%	85.9%	0.11
210	94.9%	88.5%	0.15

* Bold indicates statistical significance at α = 0.05.

*CP*, community paramedicine; *ED*, emergency department.

**Table 3:** Median healthcare utilization count per patient by cohort and time from discharge.

Days post discharge	CP cohort (N = 78)	Control cohort (N = 78)	Incidence rate ratio	95% confidence interval
Hospital readmissions				
Median (IQR)				
30	0 (0)	0 (1)	0.53	**0.30**–**0.94**
120	0 (1)	1 (2)	0.52	**0.37**–**0.74**
210	0 (1)	1 (1)	0.59	**0.45**–**0.78**
ED visits				
Median (IQR)				
30	0 (0)	0 (1)	0.50	**0.30**–**0.82**
120	0 (1)	1 (2)	0.54	**0.40**–**0.73**
210	1 (2)	1 (2)	0.59	**0.46**–**0.76**
Clinic visits				
Median (IQR)				
30	2 (2)	1 (3)	1.21	0.97–1.50
120	4 (5)	3 (7)	1.07	0.93–1.24
210	6 (7)	4.5 (7)	1.09	0.96–1.23

* Bold indicates statistical significance at α = 0.05.

*CP*, community paramedicine; *ED*, emergency department; *IQR*, interquartile range.

**Table 4:** Healthcare utilization event total count by cohort and time from discharge.

Days post discharge	CP cohort (N = 78)	Control cohort (N = 78)
Hospital readmissions		
30	18	34
120	49	94
210	81	137
ED visits		
30	23	46
120	62	115
210	102	172
Clinic visits		
30	175	145
120	389	363
210	536	492
